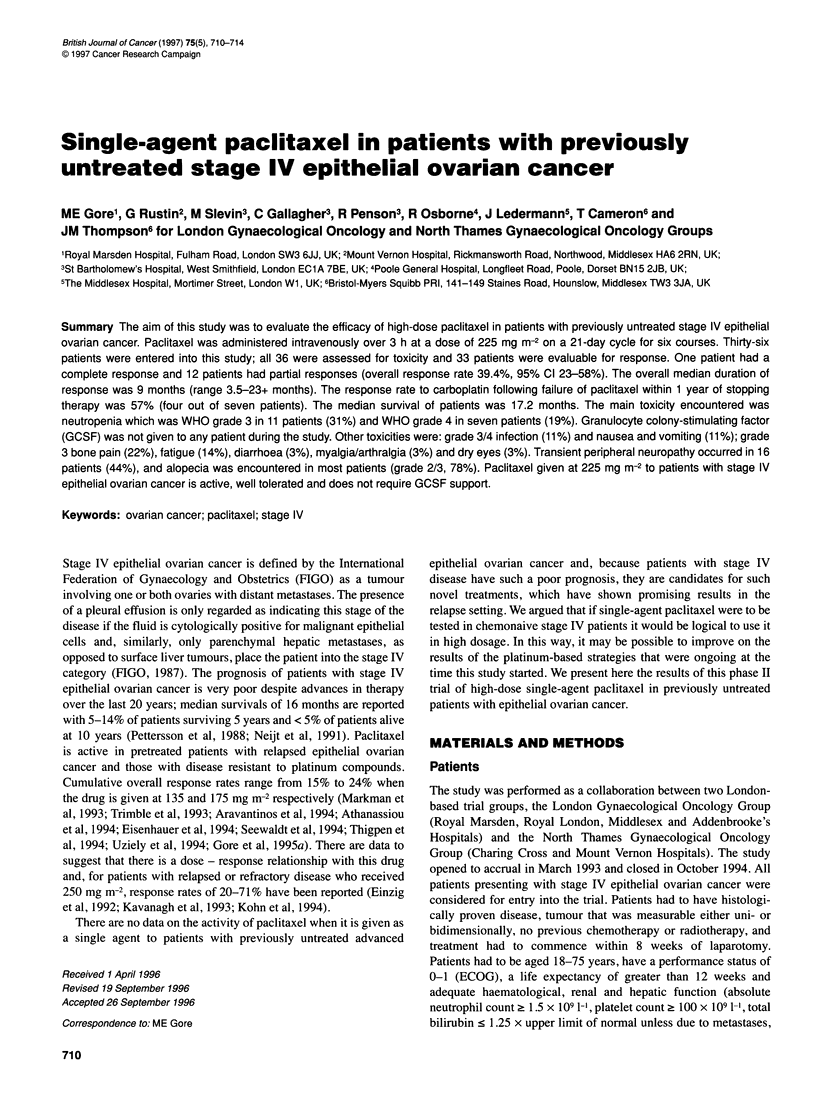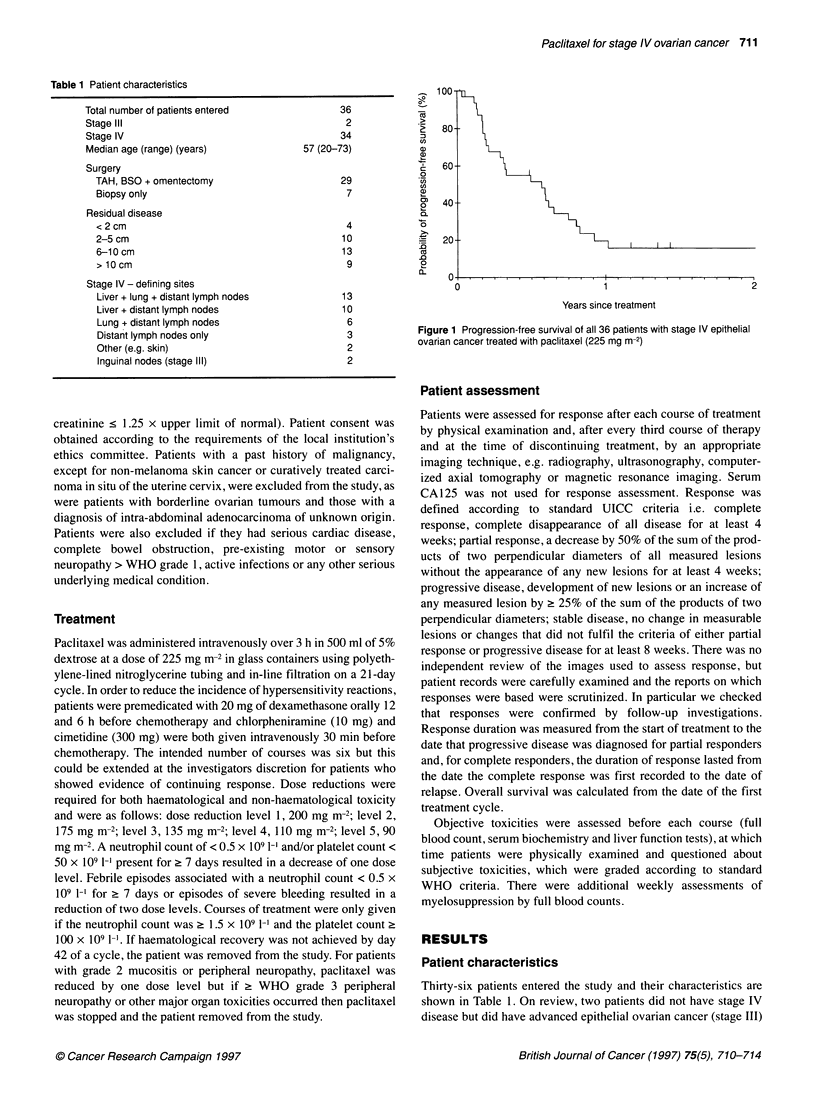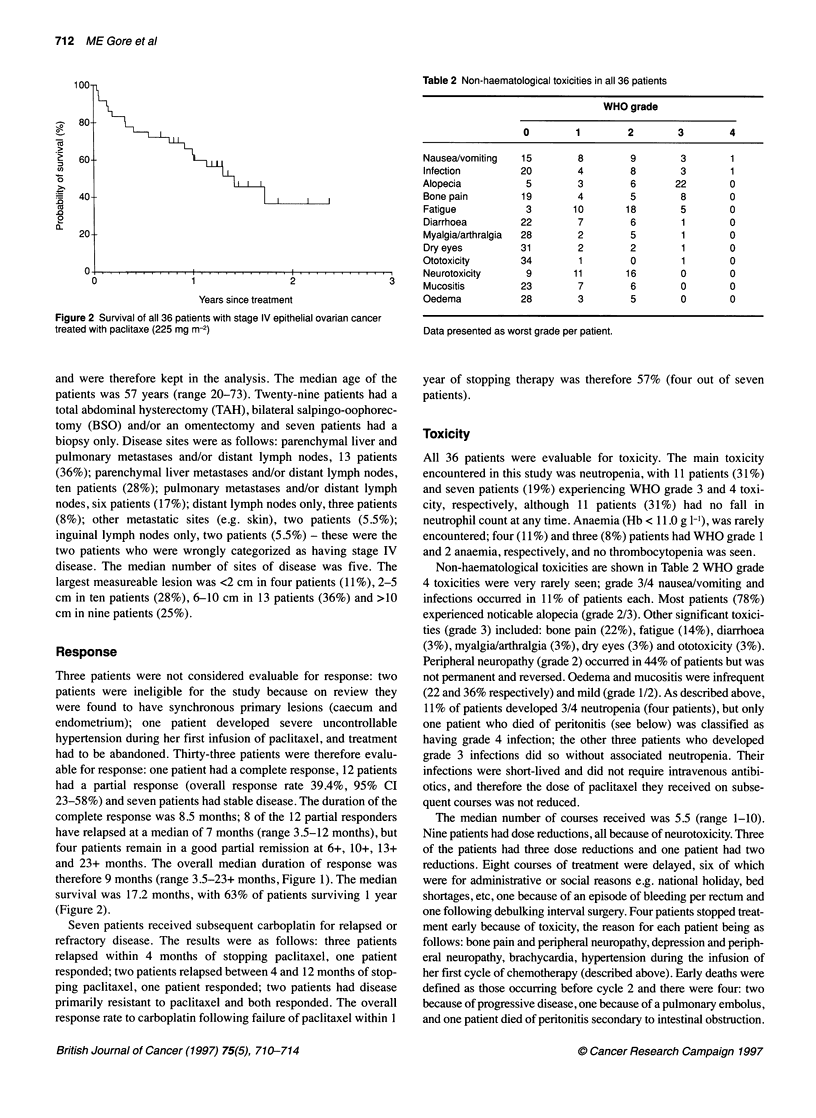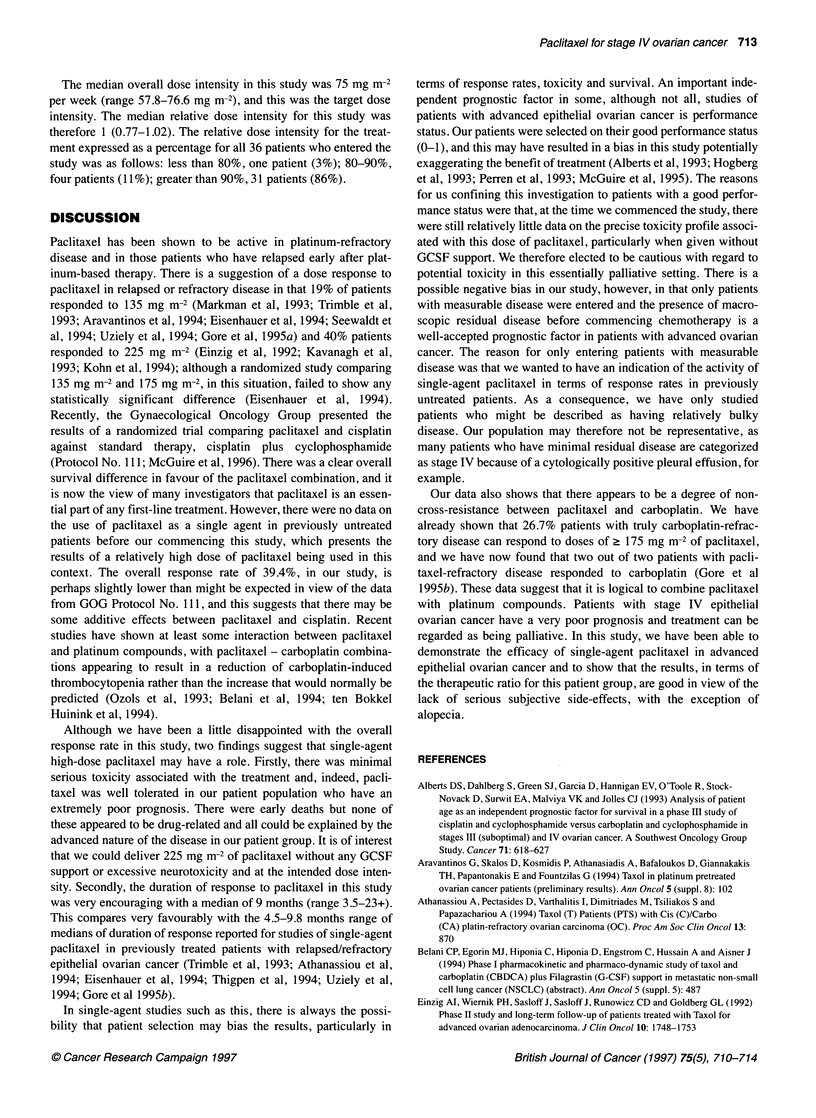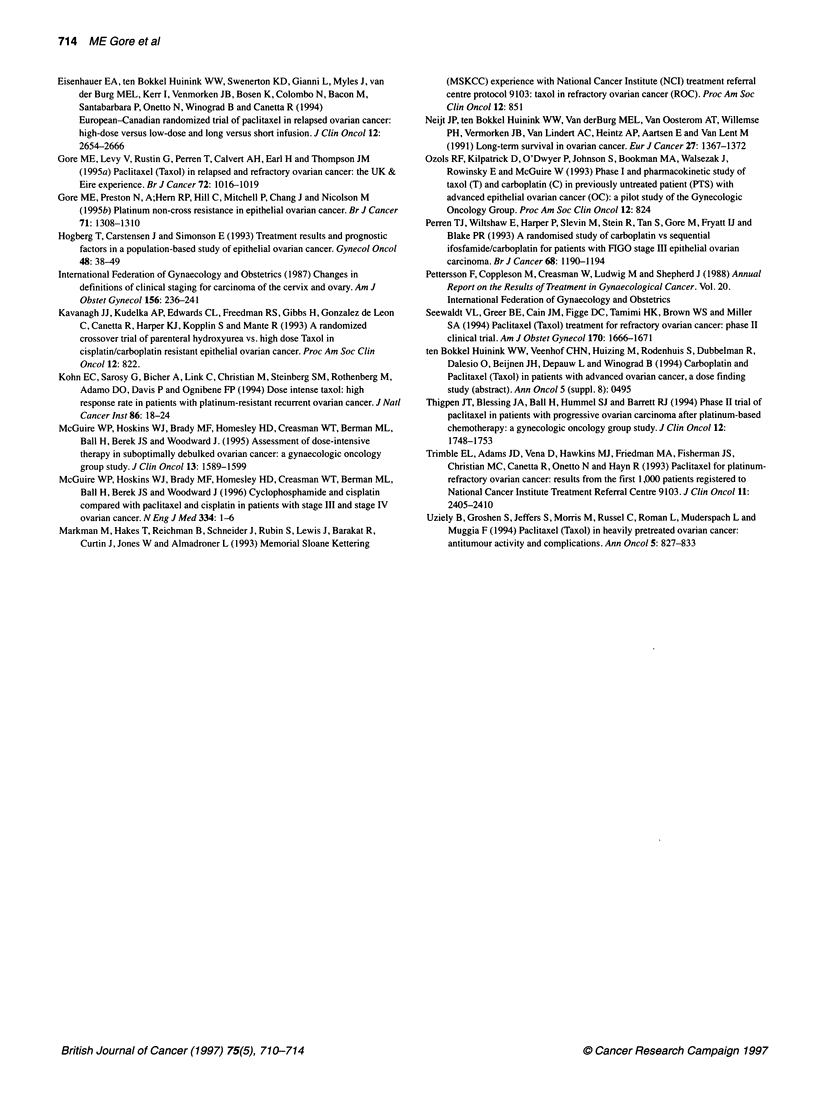# Single-agent paclitaxel in patients with previously untreated stage IV epithelial ovarian cancer. London Gynaecological Oncology and North Thames Gynaecological Oncology Groups.

**DOI:** 10.1038/bjc.1997.126

**Published:** 1997

**Authors:** M. E. Gore, G. Rustin, M. Slevin, C. Gallagher, R. Penson, R. Osborne, J. Ledermann, T. Cameron, J. M. Thompson

**Affiliations:** Royal Marsden Hospital, London, UK.

## Abstract

The aim of this study was to evaluate the efficacy of high-dose paclitaxel in patients with previously untreated stage IV epithelial ovarian cancer. Paclitaxel was administered intravenously over 3 h at a dose of 225 mg m(-2) on a 21-day cycle for six courses. Thirty-six patients were entered into this study; all 36 were assessed for toxicity and 33 patients were evaluable for response. One patient had a complete response and 12 patients had partial responses (overall response rate 39.4%, 95% CI 23-58%). The overall median duration of response was 9 months (range 3.5-23+ months). The response rate to carboplatin following failure of paclitaxel within 1 year of stopping therapy was 57% (four out of seven patients). The median survival of patients was 17.2 months. The main toxicity encountered was neutropenia which was WHO grade 3 in 11 patients (31%) and WHO grade 4 in seven patients (19%). Granulocyte colony-stimulating factor (GCSF) was not given to any patient during the study. Other toxicities were: grade 3/4 infection (11%) and nausea and vomiting (11%); grade 3 bone pain (22%), fatigue (14%), diarrhoea (3%), myalgia/arthralgia (3%) and dry eyes (3%). Transient peripheral neuropathy occurred in 16 patients (44%), and alopecia was encountered in most patients (grade 2/3, 78%). Paclitaxel given at 225 mg m(-2) to patients with stage IV epithelial ovarian cancer is active, well tolerated and does not require GCSF support.


British Journal of Cancer (1997) 75(5), 710–714
© 1997 Cancer Research Campaign

### Single-agent paclitaxel in patients with previously untreated stage IV epithelial ovarian cancer

**ME Gore[1], G Rustin[2], M Slevin[3], C Gallagher[3], R Penson[3], R Osborne[4], J Ledermann[5], T Cameron[6] and JM Thompson[6] for London Gynaecological Oncology and North Thames Gynaecological Oncology Groups**

[1]Royal Marsden Hospital, Fulham Road, London SW3 6JJ, UK; [2]Mount Vernon Hospital, Rickmansworth Road, Northwood, Middlesex HA6 2RN, UK; [3]St Bartholomew's Hospital, West Smithfield, London EC1A 7BE, UK; [4]Poole General Hospital, Longfleet Road, Poole, Dorset BN15 2JB, UK; [5]The Middlesex Hospital, Mortimer Street, London W1, UK; [6]Bristol-Myers Squibb PRI, 141–149 Staines Road, Hounslow, Middlesex TW3 3JA, UK

**Summary** The aim of this study was to evaluate the efficacy of high-dose paclitaxel in patients with previously untreated stage IV epithelial ovarian cancer. Paclitaxel was administered intravenously over 3 h at a dose of 225 mg m$^{-2}$ on a 21-day cycle for six courses. Thirty-six patients were entered into this study; all 36 were assessed for toxicity and 33 patients were evaluable for response. One patient had a complete response and 12 patients had partial responses (overall response rate 39.4%, 95% CI 23–58%). The overall median duration of response was 9 months (range 3.5–23+ months). The response rate to carboplatin following failure of paclitaxel within 1 year of stopping therapy was 57% (four out of seven patients). The median survival of patients was 17.2 months. The main toxicity encountered was neutropenia which was WHO grade 3 in 11 patients (31%) and WHO grade 4 in seven patients (19%). Granulocyte colony-stimulating factor (GCSF) was not given to any patient during the study. Other toxicities were: grade 3/4 infection (11%) and nausea and vomiting (11%); grade 3 bone pain (22%), fatigue (14%), diarrhoea (3%), myalgia/arthralgia (3%) and dry eyes (3%). Transient peripheral neuropathy occurred in 16 patients (44%), and alopecia was encountered in most patients (grade 2/3, 78%). Paclitaxel given at 225 mg m$^{-2}$ to patients with stage IV epithelial ovarian cancer is active, well tolerated and does not require GCSF support.

**Keywords:** ovarian cancer; paclitaxel; stage IV

Stage IV epithelial ovarian cancer is defined by the International Federation of Gynaecology and Obstetrics (FIGO) as a tumour involving one or both ovaries with distant metastases. The presence of a pleural effusion is only regarded as indicating this stage of the disease if the fluid is cytologically positive for malignant epithelial cells and, similarly, only parenchymal hepatic metastases, as opposed to surface liver tumours, place the patient into the stage IV category (FIGO, 1987). The prognosis of patients with stage IV epithelial ovarian cancer is very poor despite advances in therapy over the last 20 years; median survivals of 16 months are reported with 5–14% of patients surviving 5 years and < 5% of patients alive at 10 years (Pettersson et al, 1988; Neijt et al, 1991). Paclitaxel is active in pretreated patients with relapsed epithelial ovarian cancer and those with disease resistant to platinum compounds. Cumulative overall response rates range from 15% to 24% when the drug is given at 135 and 175 mg m$^{-2}$ respectively (Markman et al, 1993; Trimble et al, 1993; Aravantinos et al, 1994; Athanassiou et al, 1994; Eisenhauer et al, 1994; Seewaldt et al, 1994; Thigpen et al, 1994; Uziely et al, 1994; Gore et al, 1995*a*). There are data to suggest that there is a dose – response relationship with this drug and, for patients with relapsed or refractory disease who received 250 mg m$^{-2}$, response rates of 20–71% have been reported (Einzig et al, 1992; Kavanagh et al, 1993; Kohn et al, 1994).

There are no data on the activity of paclitaxel when it is given as a single agent to patients with previously untreated advanced epithelial ovarian cancer and, because patients with stage IV disease have such a poor prognosis, they are candidates for such novel treatments, which have shown promising results in the relapse setting. We argued that if single-agent paclitaxel were to be tested in chemonaive stage IV patients it would be logical to use it in high dosage. In this way, it may be possible to improve on the results of the platinum-based strategies that were ongoing at the time this study started. We present here the results of this phase II trial of high-dose single-agent paclitaxel in previously untreated patients with epithelial ovarian cancer.

*Received 1 April 1996*
*Revised 19 September 1996*
*Accepted 26 September 1996*

*Correspondence to:* ME Gore

### MATERIALS AND METHODS

### Patients

The study was performed as a collaboration between two London-based trial groups, the London Gynaecological Oncology Group (Royal Marsden, Royal London, Middlesex and Addenbrooke's Hospitals) and the North Thames Gynaecological Oncology Group (Charing Cross and Mount Vernon Hospitals). The study opened to accrual in March 1993 and closed in October 1994. All patients presenting with stage IV epithelial ovarian cancer were considered for entry into the trial. Patients had to have histologically proven disease, tumour that was measurable either uni- or bidimensionally, no previous chemotherapy or radiotherapy, and treatment had to commence within 8 weeks of laparotomy. Patients had to be aged 18–75 years, have a performance status of 0–1 (ECOG), a life expectancy of greater than 12 weeks and adequate haematological, renal and hepatic function (absolute neutrophil count ≥ $1.5 \times 10^9$ l$^{-1}$, platelet count ≥ $100 \times 10^9$ l$^{-1}$, total bilirubin ≤ 1.25 × upper limit of normal unless due to metastases,

**Table 1** Patient characteristics

| | |
|---|---|
| Total number of patients entered | 36 |
| Stage III | 2 |
| Stage IV | 34 |
| Median age (range) (years) | 57 (20–73) |
| Surgery | |
| TAH, BSO + omentectomy | 29 |
| Biopsy only | 7 |
| Residual disease | |
| < 2 cm | 4 |
| 2–5 cm | 10 |
| 6–10 cm | 13 |
| > 10 cm | 9 |
| Stage IV – defining sites | |
| Liver + lung + distant lymph nodes | 13 |
| Liver + distant lymph nodes | 10 |
| Lung + distant lymph nodes | 6 |
| Distant lymph nodes only | 3 |
| Other (e.g. skin) | 2 |
| Inguinal nodes (stage III) | 2 |

creatinine ≤ 1.25 × upper limit of normal). Patient consent was obtained according to the requirements of the local institution's ethics committee. Patients with a past history of malignancy, except for non-melanoma skin cancer or curatively treated carcinoma in situ of the uterine cervix, were excluded from the study, as were patients with borderline ovarian tumours and those with a diagnosis of intra-abdominal adenocarcinoma of unknown origin. Patients were also excluded if they had serious cardiac disease, complete bowel obstruction, pre-existing motor or sensory neuropathy > WHO grade 1, active infections or any other serious underlying medical condition.

### Treatment

Paclitaxel was administered intravenously over 3 h in 500 ml of 5% dextrose at a dose of 225 mg $m^{-2}$ in glass containers using polyethylene-lined nitroglycerine tubing and in-line filtration on a 21-day cycle. In order to reduce the incidence of hypersensitivity reactions, patients were premedicated with 20 mg of dexamethasone orally 12 and 6 h before chemotherapy and chlorpheniramine (10 mg) and cimetidine (300 mg) were both given intravenously 30 min before chemotherapy. The intended number of courses was six but this could be extended at the investigators discretion for patients who showed evidence of continuing response. Dose reductions were required for both haematological and non-haematological toxicity and were as follows: dose reduction level 1, 200 mg $m^{-2}$; level 2, 175 mg $m^{-2}$; level 3, 135 mg $m^{-2}$; level 4, 110 mg $m^{-2}$; level 5, 90 mg $m^{-2}$. A neutrophil count of $< 0.5 \times 10^9\ l^{-1}$ and/or platelet count $< 50 \times 10^9\ l^{-1}$ present for ≥ 7 days resulted in a decrease of one dose level. Febrile episodes associated with a neutrophil count $< 0.5 \times 10^9\ l^{-1}$ for ≥ 7 days or episodes of severe bleeding resulted in a reduction of two dose levels. Courses of treatment were only given if the neutrophil count was $\geq 1.5 \times 10^9\ l^{-1}$ and the platelet count $\geq 100 \times 10^9\ l^{-1}$. If haematological recovery was not achieved by day 42 of a cycle, the patient was removed from the study. For patients with grade 2 mucositis or peripheral neuropathy, paclitaxel was reduced by one dose level but if ≥ WHO grade 3 peripheral neuropathy or other major organ toxicities occurred then paclitaxel was stopped and the patient removed from the study.

**Figure 1** Progression-free survival of all 36 patients with stage IV epithelial ovarian cancer treated with paclitaxel (225 mg $m^{-2}$)

### Patient assessment

Patients were assessed for response after each course of treatment by physical examination and, after every third course of therapy and at the time of discontinuing treatment, by an appropriate imaging technique, e.g. radiography, ultrasonography, computerized axial tomography or magnetic resonance imaging. Serum CA125 was not used for response assessment. Response was defined according to standard UICC criteria i.e. complete response, complete disappearance of all disease for at least 4 weeks; partial response, a decrease by 50% of the sum of the products of two perpendicular diameters of all measured lesions without the appearance of any new lesions for at least 4 weeks; progressive disease, development of new lesions or an increase of any measured lesion by ≥ 25% of the sum of the products of two perpendicular diameters; stable disease, no change in measurable lesions or changes that did not fulfil the criteria of either partial response or progressive disease for at least 8 weeks. There was no independent review of the images used to assess response, but patient records were carefully examined and the reports on which responses were based were scrutinized. In particular we checked that responses were confirmed by follow-up investigations. Response duration was measured from the start of treatment to the date that progressive disease was diagnosed for partial responders and, for complete responders, the duration of response lasted from the date the complete response was first recorded to the date of relapse. Overall survival was calculated from the date of the first treatment cycle.

Objective toxicities were assessed before each course (full blood count, serum biochemistry and liver function tests), at which time patients were physically examined and questioned about subjective toxicities, which were graded according to standard WHO criteria. There were additional weekly assessments of myelosuppression by full blood counts.

### RESULTS

### Patient characteristics

Thirty-six patients entered the study and their characteristics are shown in Table 1. On review, two patients did not have stage IV disease but did have advanced epithelial ovarian cancer (stage III)

© Cancer Research Campaign 1997

**Figure 2** Survival of all 36 patients with stage IV epithelial ovarian cancer treated with paclitaxe (225 mg m-2)

**Table 2** Non-haematological toxicities in all 36 patients

| | WHO grade | | | | |
|---|---|---|---|---|---|
| | 0 | 1 | 2 | 3 | 4 |
| Nausea/vomiting | 15 | 8 | 9 | 3 | 1 |
| Infection | 20 | 4 | 8 | 3 | 1 |
| Alopecia | 5 | 3 | 6 | 22 | 0 |
| Bone pain | 19 | 4 | 5 | 8 | 0 |
| Fatigue | 3 | 10 | 18 | 5 | 0 |
| Diarrhoea | 22 | 7 | 6 | 1 | 0 |
| Myalgia/arthralgia | 28 | 2 | 5 | 1 | 0 |
| Dry eyes | 31 | 2 | 2 | 1 | 0 |
| Ototoxicity | 34 | 1 | 0 | 1 | 0 |
| Neurotoxicity | 9 | 11 | 16 | 0 | 0 |
| Mucositis | 23 | 7 | 6 | 0 | 0 |
| Oedema | 28 | 3 | 5 | 0 | 0 |

Data presented as worst grade per patient.

and were therefore kept in the analysis. The median age of the patients was 57 years (range 20–73). Twenty-nine patients had a total abdominal hysterectomy (TAH), bilateral salpingo-oophorectomy (BSO) and/or an omentectomy and seven patients had a biopsy only. Disease sites were as follows: parenchymal liver and pulmonary metastases and/or distant lymph nodes, 13 patients (36%); parenchymal liver metastases and/or distant lymph nodes, ten patients (28%); pulmonary metastases and/or distant lymph nodes, six patients (17%); distant lymph nodes only, three patients (8%); other metastatic sites (e.g. skin), two patients (5.5%); inguinal lymph nodes only, two patients (5.5%) – these were the two patients who were wrongly categorized as having stage IV disease. The median number of sites of disease was five. The largest measureable lesion was <2 cm in four patients (11%), 2–5 cm in ten patients (28%), 6–10 cm in 13 patients (36%) and >10 cm in nine patients (25%).

### Response

Three patients were not considered evaluable for response: two patients were ineligible for the study because on review they were found to have synchronous primary lesions (caecum and endometrium); one patient developed severe uncontrollable hypertension during her first infusion of paclitaxel, and treatment had to be abandoned. Thirty-three patients were therefore evaluable for response: one patient had a complete response, 12 patients had a partial response (overall response rate 39.4%, 95% CI 23–58%) and seven patients had stable disease. The duration of the complete response was 8.5 months; 8 of the 12 partial responders have relapsed at a median of 7 months (range 3.5–12 months), but four patients remain in a good partial remission at 6+, 10+, 13+ and 23+ months. The overall median duration of response was therefore 9 months (range 3.5–23+ months, Figure 1). The median survival was 17.2 months, with 63% of patients surviving 1 year (Figure 2).

Seven patients received subsequent carboplatin for relapsed or refractory disease. The results were as follows: three patients relapsed within 4 months of stopping paclitaxel, one patient responded; two patients relapsed between 4 and 12 months of stopping paclitaxel, one patient responded; two patients had disease primarily resistant to paclitaxel and both responded. The overall response rate to carboplatin following failure of paclitaxel within 1 year of stopping therapy was therefore 57% (four out of seven patients).

### Toxicity

All 36 patients were evaluable for toxicity. The main toxicity encountered in this study was neutropenia, with 11 patients (31%) and seven patients (19%) experiencing WHO grade 3 and 4 toxicity, respectively, although 11 patients (31%) had no fall in neutrophil count at any time. Anaemia (Hb < 11.0 g $l^{-1}$), was rarely encountered; four (11%) and three (8%) patients had WHO grade 1 and 2 anaemia, respectively, and no thrombocytopenia was seen.

Non-haematological toxicities are shown in Table 2 WHO grade 4 toxicities were very rarely seen; grade 3/4 nausea/vomiting and infections occurred in 11% of patients each. Most patients (78%) experienced noticable alopecia (grade 2/3). Other significant toxicities (grade 3) included: bone pain (22%), fatigue (14%), diarrhoea (3%), myalgia/arthralgia (3%), dry eyes (3%) and ototoxicity (3%). Peripheral neuropathy (grade 2) occurred in 44% of patients but was not permanent and reversed. Oedema and mucositis were infrequent (22 and 36% respectively) and mild (grade 1/2). As described above, 11% of patients developed 3/4 neutropenia (four patients), but only one patient who died of peritonitis (see below) was classified as having grade 4 infection; the other three patients who developed grade 3 infections did so without associated neutropenia. Their infections were short-lived and did not require intravenous antibiotics, and therefore the dose of paclitaxel they received on subsequent courses was not reduced.

The median number of courses received was 5.5 (range 1–10). Nine patients had dose reductions, all because of neurotoxicity. Three of the patients had three dose reductions and one patient had two reductions. Eight courses of treatment were delayed, six of which were for administrative or social reasons e.g. national holiday, bed shortages, etc, one because of an episode of bleeding per rectum and one following debulking interval surgery. Four patients stopped treatment early because of toxicity, the reason for each patient being as follows: bone pain and peripheral neuropathy, depression and peripheral neuropathy, brachycardia, hypertension during the infusion of her first cycle of chemotherapy (described above). Early deaths were defined as those occurring before cycle 2 and there were four: two because of progressive disease, one because of a pulmonary embolus, and one patient died of peritonitis secondary to intestinal obstruction.

© Cancer Research Campaign 1997

The median overall dose intensity in this study was 75 mg $m^{-2}$ per week (range 57.8–76.6 mg $m^{-2}$), and this was the target dose intensity. The median relative dose intensity for this study was therefore 1 (0.77–1.02). The relative dose intensity for the treatment expressed as a percentage for all 36 patients who entered the study was as follows: less than 80%, one patient (3%); 80–90%, four patients (11%); greater than 90%, 31 patients (86%).

### DISCUSSION

Paclitaxel has been shown to be active in platinum-refractory disease and in those patients who have relapsed early after platinum-based therapy. There is a suggestion of a dose response to paclitaxel in relapsed or refractory disease in that 19% of patients responded to 135 mg $m^{-2}$ (Markman et al, 1993; Trimble et al, 1993; Aravantinos et al, 1994; Eisenhauer et al, 1994; Seewaldt et al, 1994; Uziely et al, 1994; Gore et al, 1995*a*) and 40% patients responded to 225 mg $m^{-2}$ (Einzig et al, 1992; Kavanagh et al, 1993; Kohn et al, 1994); although a randomized study comparing 135 mg $m^{-2}$ and 175 mg $m^{-2}$, in this situation, failed to show any statistically significant difference (Eisenhauer et al, 1994). Recently, the Gynaecological Oncology Group presented the results of a randomized trial comparing paclitaxel and cisplatin against standard therapy, cisplatin plus cyclophosphamide (Protocol No. 111; McGuire et al, 1996). There was a clear overall survival difference in favour of the paclitaxel combination, and it is now the view of many investigators that paclitaxel is an essential part of any first-line treatment. However, there were no data on the use of paclitaxel as a single agent in previously untreated patients before our commencing this study, which presents the results of a relatively high dose of paclitaxel being used in this context. The overall response rate of 39.4%, in our study, is perhaps slightly lower than might be expected in view of the data from GOG Protocol No. 111, and this suggests that there may be some additive effects between paclitaxel and cisplatin. Recent studies have shown at least some interaction between paclitaxel and platinum compounds, with paclitaxel – carboplatin combinations appearing to result in a reduction of carboplatin-induced thrombocytopenia rather than the increase that would normally be predicted (Ozols et al, 1993; Belani et al, 1994; ten Bokkel Huinink et al, 1994).

Although we have been a little disappointed with the overall response rate in this study, two findings suggest that single-agent high-dose paclitaxel may have a role. Firstly, there was minimal serious toxicity associated with the treatment and, indeed, paclitaxel was well tolerated in our patient population who have an extremely poor prognosis. There were early deaths but none of these appeared to be drug-related and all could be explained by the advanced nature of the disease in our patient group. It is of interest that we could deliver 225 mg $m^{-2}$ of paclitaxel without any GCSF support or excessive neurotoxicity and at the intended dose intensity. Secondly, the duration of response to paclitaxel in this study was very encouraging with a median of 9 months (range 3.5–23+). This compares very favourably with the 4.5–9.8 months range of medians of duration of response reported for studies of single-agent paclitaxel in previously treated patients with relapsed/refractory epithelial ovarian cancer (Trimble et al, 1993; Athanassiou et al, 1994; Eisenhauer et al, 1994; Thigpen et al, 1994; Uziely et al, 1994; Gore et al 1995*b*).

In single-agent studies such as this, there is always the possibility that patient selection may bias the results, particularly in terms of response rates, toxicity and survival. An important independent prognostic factor in some, although not all, studies of patients with advanced epithelial ovarian cancer is performance status. Our patients were selected on their good performance status (0–1), and this may have resulted in a bias in this study potentially exaggerating the benefit of treatment (Alberts et al, 1993; Hogberg et al, 1993; Perren et al, 1993; McGuire et al, 1995). The reasons for us confining this investigation to patients with a good performance status were that, at the time we commenced the study, there were still relatively little data on the precise toxicity profile associated with this dose of paclitaxel, particularly when given without GCSF support. We therefore elected to be cautious with regard to potential toxicity in this essentially palliative setting. There is a possible negative bias in our study, however, in that only patients with measurable disease were entered and the presence of macroscopic residual disease before commencing chemotherapy is a well-accepted prognostic factor in patients with advanced ovarian cancer. The reason for only entering patients with measurable disease was that we wanted to have an indication of the activity of single-agent paclitaxel in terms of response rates in previously untreated patients. As a consequence, we have only studied patients who might be described as having relatively bulky disease. Our population may therefore not be representative, as many patients who have minimal residual disease are categorized as stage IV because of a cytologically positive pleural effusion, for example.

Our data also shows that there appears to be a degree of non-cross-resistance between paclitaxel and carboplatin. We have already shown that 26.7% patients with truly carboplatin-refractory disease can respond to doses of ≥ 175 mg $m^{-2}$ of paclitaxel, and we have now found that two out of two patients with paclitaxel-refractory disease responded to carboplatin (Gore et al 1995*b*). These data suggest that it is logical to combine paclitaxel with platinum compounds. Patients with stage IV epithelial ovarian cancer have a very poor prognosis and treatment can be regarded as being palliative. In this study, we have been able to demonstrate the efficacy of single-agent paclitaxel in advanced epithelial ovarian cancer and to show that the results, in terms of the therapeutic ratio for this patient group, are good in view of the lack of serious subjective side-effects, with the exception of alopecia.

### REFERENCES

Alberts DS, Dahlberg S, Green SJ, Garcia D, Hannigan EV, O'Toole R, Stock-Novack D, Surwit EA, Malviya VK and Jolles CJ (1993) Analysis of patient age as an independent prognostic factor for survival in a phase III study of cisplatin and cyclophosphamide versus carboplatin and cyclophosphamide in stages III (suboptimal) and IV ovarian cancer. A Southwest Oncology Group Study. *Cancer* **71**: 618–627

Aravantinos G, Skalos D, Kosmidis P, Athanasiadis A, Bafaloukos D, Giannakakis TH, Papantonakis E and Fountzilas G (1994) Taxol in platinum pretreated ovarian cancer patients (preliminary results). *Ann Oncol* **5** (suppl. 8): 102

Athanassiou A, Pectasides D, Varthalitis I, Dimitriades M, Tsiliakos S and Papazachariou A (1994) Taxol (T) Patients (PTS) with Cis (C)/Carbo (CA) platin-refractory ovarian carcinoma (OC). *Proc Am Soc Clin Oncol* **13**: 870

Belani CP, Egorin MJ, Hiponia C, Hiponia D, Engstrom C, Hussain A and Aisner J (1994) Phase I pharmacokinetic and pharmaco-dynamic study of taxol and carboplatin (CBDCA) plus Filagrastin (G-CSF) support in metastatic non-small cell lung cancer (NSCLC) (abstract). *Ann Oncol* 5 (suppl. 5): 487

Einzig AI, Wiernik PH, Sasloff J, Sasloff J, Runowicz CD and Goldberg GL (1992) Phase II study and long-term follow-up of patients treated with Taxol for advanced ovarian adenocarcinoma. *J Clin Oncol* **10**: 1748–1753

Eisenhauer EA, ten Bokkel Huinink WW, Swenerton KD, Gianni L, Myles J, van der Burg MEL, Kerr I, Venmorken JB, Bosen K, Colombo N, Bacon M, Santabarbara P, Onetto N, Winograd B and Canetta R (1994) European–Canadian randomized trial of paclitaxel in relapsed ovarian cancer: high-dose versus low-dose and long versus short infusion. *J Clin Oncol* **12**: 2654–2666

Gore ME, Levy V, Rustin G, Perren T, Calvert AH, Earl H and Thompson JM (1995*a*) Paclitaxel (Taxol) in relapsed and refractory ovarian cancer: the UK & Eire experience. *Br J Cancer* **72**: 1016–1019

Gore ME, Preston N, A;Hern RP, Hill C, Mitchell P, Chang J and Nicolson M (1995*b*) Platinum non-cross resistance in epithelial ovarian cancer. *Br J Cancer* **71**: 1308–1310

Hogberg T, Carstensen J and Simonson E (1993) Treatment results and prognostic factors in a population-based study of epithelial ovarian cancer. *Gynecol Oncol* **48**: 38–49

International Federation of Gynaecology and Obstetrics (1987) Changes in definitions of clinical staging for carcinoma of the cervix and ovary. *Am J Obstet Gynecol* **156**: 236–241

Kavanagh JJ, Kudelka AP, Edwards CL, Freedman RS, Gibbs H, Gonzalez de Leon C, Canetta R, Harper KJ, Kopplin S and Mante R (1993) A randomized crossover trial of parenteral hydroxyurea vs. high dose Taxol in cisplatin/carboplatin resistant epithelial ovarian cancer. *Proc Am Soc Clin Oncol* **12**: 822.

Kohn EC, Sarosy G, Bicher A, Link C, Christian M, Steinberg SM, Rothenberg M, Adamo DO, Davis P and Ognibene FP (1994) Dose intense taxol: high response rate in patients with platinum-resistant recurrent ovarian cancer. *J Natl Cancer Inst* **86**: 18–24

McGuire WP, Hoskins WJ, Brady MF, Homesley HD, Creasman WT, Berman ML, Ball H, Berek JS and Woodward J. (1995) Assessment of dose-intensive therapy in suboptimally debulked ovarian cancer: a gynaecologic oncology group study. *J Clin Oncol* **13**: 1589–1599

McGuire WP, Hoskins WJ, Brady MF, Homesley HD, Creasman WT, Berman ML, Ball H, Berek JS and Woodward J (1996) Cyclophosphamide and cisplatin compared with paclitaxel and cisplatin in patients with stage III and stage IV ovarian cancer. *N Eng J Med* **334**: 1–6

Markman M, Hakes T, Reichman B, Schneider J, Rubin S, Lewis J, Barakat R, Curtin J, Jones W and Almadroner L (1993) Memorial Sloane Kettering (MSKCC) experience with National Cancer Institute (NCI) treatment referral centre protocol 9103: taxol in refractory ovarian cancer (ROC). *Proc Am Soc Clin Oncol* **12**: 851

Neijt JP, ten Bokkel Huinink WW, Van derBurg MEL, Van Oosterom AT, Willemse PH, Vermorken JB, Van Lindert AC, Heintz AP, Aartsen E and Van Lent M (1991) Long-term survival in ovarian cancer. *Eur J Cancer* **27**: 1367–1372

Ozols RF, Kilpatrick D, O'Dwyer P, Johnson S, Bookman MA, Walsezak J, Rowinsky E and McGuire W (1993) Phase I and pharmacokinetic study of taxol (T) and carboplatin (C) in previously untreated patient (PTS) with advanced epithelial ovarian cancer (OC): a pilot study of the Gynecologic Oncology Group. *Proc Am Soc Clin Oncol* **12**: 824

Perren TJ, Wiltshaw E, Harper P, Slevin M, Stein R, Tan S, Gore M, Fryatt IJ and Blake PR (1993) A randomised study of carboplatin vs sequential ifosfamide/carboplatin for patients with FIGO stage III epithelial ovarian carcinoma. *Br J Cancer* **68**: 1190–1194

Pettersson F, Coppleson M, Creasman W, Ludwig M and Shepherd J (1988) *Annual Report on the Results of Treatment in Gynaecological Cancer*. Vol. 20. International Federation of Gynaecology and Obstetrics

Seewaldt VL, Greer BE, Cain JM, Figge DC, Tamimi HK, Brown WS and Miller SA (1994) Paclitaxel (Taxol) treatment for refractory ovarian cancer: phase II clinical trial. *Am J Obstet Gynecol* **170**: 1666–1671

ten Bokkel Huinink WW, Veenhof CHN, Huizing M, Rodenhuis S, Dubbelman R, Dalesio O, Beijnen JH, Depauw L and Winograd B (1994) Carboplatin and Paclitaxel (Taxol) in patients with advanced ovarian cancer, a dose finding study (abstract). *Ann Oncol* 5 (suppl. 8): 0495

Thigpen JT, Blessing JA, Ball H, Hummel SJ and Barrett RJ (1994) Phase II trial of paclitaxel in patients with progressive ovarian carcinoma after platinum-based chemotherapy: a gynecologic oncology group study. *J Clin Oncol* **12**: 1748–1753

Trimble EL, Adams JD, Vena D, Hawkins MJ, Friedman MA, Fisherman JS, Christian MC, Canetta R, Onetto N and Hayn R (1993) Paclitaxel for platinum-refractory ovarian cancer: results from the first 1,000 patients registered to National Cancer Institute Treatment Referral Centre 9103. *J Clin Oncol* **11**: 2405–2410

Uziely B, Groshen S, Jeffers S, Morris M, Russel C, Roman L, Muderspach L and Muggia F (1994) Paclitaxel (Taxol) in heavily pretreated ovarian cancer: antitumour activity and complications. *Ann Oncol* **5**: 827–833

© Cancer Research Campaign 1997